# Supplementary material for: Healthy behaviours, treatment, and control status of diagnosed hypertension and diabetes among the government nurses and para-health professionals of Bangladesh: A cross-sectional study
Source: PLOS Glob Public Health. 2023 Aug 1;3(8):e0002234. doi: 10.1371/journal.pgph.0002234 (PMC10393152; doi:10.1371/journal.pgph.0002234)
Supplement: S1 Data — (PDF) [file pgph.0002234.s001.pdf]

S1_Data: Multivariate and univariate outlier detection

Multivariate outlier detection using Mahalanobis Distance (MD) Method

Serial	Study member ID	Mahalanobis Distance	*P*-value	Outlier
1.	659	34.64258	.00003	Yes
2.	1360	28.04315	.00047	Yes
3.	365	27.85083	.00050	Yes
4.	194	23.94772	.00234	No
5.	671	23.16648	.00316	No
6.	1386	21.10522	.00687	No
7.	1192	20.99653	.00716	No
8.	1258	20.98978	.00717	No
9.	600	20.88795	.00745	No
10.	412	20.44781	.00877	No
11.	1587	19.93440	.01059	No
12.	1347	19.81566	.01106	No
13.	948	17.70658	.02354	No
14.	1586	17.29860	.02715	No
15.	259	17.27796	.02734	No
16.	1544	16.56509	.03497	No
17.	1875	16.27723	.03858	No
18.	672	15.91484	.04362	No
19.	1560	15.62060	.04814	No
20.	954	15.04124	.05835	No
21.	669	14.64353	.06646	No
22.	652	13.56134	.09394	No
23.	896	13.49652	.09587	No
24.	832	12.85110	.11708	No
25.	205	12.30407	.13814	No
26.	1198	12.20608	.14224	No
27.	1614	12.15486	.14443	No
28.	747	11.71069	.16459	No
29.	1621	11.57640	.17113	No
30.	1274	11.21908	.18959	No
31.	825	10.96269	.20382	No
32.	596	10.72239	.21793	No
33.	1922	10.62892	.22362	No
34.	1435	10.47654	.23316	No
35.	1167	10.44695	.23505	No
36.	1679	10.15130	.25457	No
37.	639	10.10299	.25787	No
38.	404	9.98931	.26578	No
39.	398	9.90947	.27144	No
40.	1736	9.71862	.28533	No
41.	693	9.56213	.29711	No
42.	1518	9.52286	.30012	No

43.	1151	9.16804	.32832	No
44.	289	9.16391	.32866	No
45.	1563	9.05447	.33772	No
46.	1388	9.03526	.33933	No
47.	1246	8.85838	.35438	No
48.	352	8.80183	.35929	No
49.	280	8.80063	.35939	No
50.	603	8.75965	.36298	No
51.	47	8.71532	.36688	No
52.	571	8.61078	.37619	No
53.	1833	8.60101	.37706	No
54.	946	8.59360	.37773	No
55.	1374	8.56477	.38033	No
56.	1189	8.54863	.38179	No
57.	903	8.50127	.38610	No
58.	35	8.45525	.39031	No
59.	166	8.41422	.39409	No
60.	1543	8.38700	.39661	No
61.	200	8.27082	.40747	No
62.	1184	8.23509	.41085	No
63.	1527	8.08737	.42498	No
64.	244	8.07702	.42598	No
65.	286	7.92428	.44090	No
66.	1772	7.91209	.44210	No
67.	1006	7.91030	.44228	No
68.	31	7.85247	.44801	No
69.	1823	7.84319	.44894	No
70.	1251	7.77634	.45562	No
71.	1814	7.65934	.46743	No
72.	881	7.65134	.46825	No
73.	1809	7.38777	.49543	No
74.	1782	7.37301	.49697	No
75.	1161	7.30911	.50368	No
76.	735	7.29048	.50564	No
77.	34	7.27650	.50712	No
78.	160	7.27357	.50742	No
79.	1707	7.19817	.51541	No
80.	486	7.18685	.51661	No
81.	675	7.18142	.51719	No
82.	1098	7.15064	.52047	No
83.	1774	7.10211	.52566	No
84.	784	7.06380	.52976	No
85.	790	7.05487	.53072	No
86.	767	7.04462	.53183	No
87.	1669	6.99273	.53742	No
88.	1359	6.95333	.54168	No

89.	716	6.95292	.54172	No
90.	282	6.92441	.54481	No
91.	1346	6.91617	.54570	No
92.	705	6.88497	.54909	No
93.	720	6.75130	.56369	No
94.	1904	6.73490	.56549	No
95.	1558	6.71206	.56799	No
96.	1903	6.69134	.57027	No
97.	1742	6.64253	.57565	No
98.	1269	6.60369	.57993	No
99.	1834	6.56010	.58475	No
100.	235	6.44960	.59700	No
101.	50	6.39577	.60299	No
102.	1589	6.32047	.61138	No
103.	376	6.29248	.61451	No
104.	664	6.21149	.62355	No
105.	1887	6.19543	.62535	No
106.	1804	6.17910	.62718	No
107.	1895	6.16678	.62856	No
108.	746	6.13540	.63207	No
109.	1174	6.11407	.63446	No
110.	1526	6.11065	.63484	No
111.	738	6.10642	.63531	No
112.	285	6.10004	.63603	No
113.	1691	6.03291	.64355	No
114.	310	6.01295	.64578	No
115.	681	5.98047	.64942	No
116.	37	5.97883	.64960	No
117.	233	5.96569	.65108	No
118.	1011	5.95063	.65276	No
119.	963	5.93212	.65484	No
120.	388	5.87783	.66091	No
121.	981	5.86868	.66194	No
122.	1905	5.74243	.67606	No
123.	38	5.61280	.69051	No
124.	79	5.61089	.69073	No
125.	1916	5.51976	.70085	No
126.	1569	5.49229	.70389	No
127.	473	5.39489	.71465	No
128.	1342	5.36773	.71765	No
129.	709	5.36339	.71812	No
130.	197	5.31455	.72349	No
131.	393	5.30809	.72420	No
132.	1142	5.26425	.72900	No
133.	1511	5.22769	.73299	No
134.	976	5.17035	.73922	No

135.	48	5.15684	.74069	No
136.	713	5.12188	.74447	No
137.	760	5.04029	.75326	No
138.	1382	5.02177	.75525	No
139.	700	5.02125	.75530	No
140.	229	4.99758	.75783	No
141.	574	4.98824	.75883	No
142.	202	4.97135	.76063	No
143.	677	4.91938	.76616	No
144.	793	4.91370	.76676	No
145.	1188	4.86602	.77180	No
146.	1585	4.86592	.77181	No
147.	1925	4.75109	.78382	No
148.	1796	4.74766	.78417	No
149.	1541	4.72745	.78627	No
150.	1876	4.68935	.79020	No
151.	193	4.65947	.79327	No
152.	1149	4.65562	.79367	No
153.	347	4.59117	.80024	No
154.	1767	4.59026	.80034	No
155.	219	4.57363	.80202	No
156.	161	4.56900	.80249	No
157.	660	4.53984	.80543	No
158.	1513	4.46551	.81287	No
159.	869	4.45642	.81378	No
160.	610	4.45292	.81412	No
161.	1902	4.38170	.82115	No
162.	147	4.37374	.82193	No
163.	196	4.35101	.82415	No
164.	43	4.34369	.82486	No
165.	1143	4.32742	.82644	No
166.	1901	4.21948	.83680	No
167.	1935	4.17272	.84121	No
168.	1423	4.17224	.84126	No
169.	214	4.11763	.84636	No
170.	1536	4.09530	.84842	No
171.	611	4.07917	.84991	No
172.	49	4.04828	.85274	No
173.	1538	3.98430	.85854	No
174.	889	3.91491	.86472	No
175.	795	3.87504	.86822	No
176.	882	3.86287	.86928	No
177.	680	3.86115	.86943	No
178.	1936	3.84428	.87089	No
179.	1345	3.78680	.87583	No
180.	1537	3.75471	.87855	No

181.	232	3.74990	.87895	No
182.	1881	3.71434	.88193	No
183.	459	3.66950	.88564	No
184.	228	3.66390	.88610	No
185.	1395	3.66045	.88639	No
186.	980	3.65652	.88671	No
187.	1827	3.64946	.88729	No
188.	1357	3.53746	.89626	No
189.	1831	3.47359	.90123	No
190.	1581	3.36158	.90966	No
191.	1826	3.35599	.91007	No
192.	692	3.28876	.91495	No
193.	657	3.28556	.91518	No
194.	1245	3.26339	.91676	No
195.	1201	3.26283	.91680	No
196.	1152	3.24561	.91801	No
197.	1545	3.20406	.92091	No
198.	676	3.19084	.92182	No
199.	960	3.15657	.92415	No
200.	1780	3.13849	.92537	No
201.	32	3.10326	.92771	No
202.	212	3.07360	.92965	No
203.	231	2.98907	.93504	No
204.	1924	2.98620	.93522	No
205.	1914	2.96076	.93680	No
206.	1692	2.94631	.93768	No
207.	1590	2.87870	.94174	No
208.	565	2.85500	.94312	No
209.	865	2.84995	.94342	No
210.	46	2.83715	.94416	No
211.	1622	2.83081	.94452	No
212.	299	2.81732	.94529	No
213.	1016	2.75167	.94896	No
214.	245	2.74714	.94921	No
215.	206	2.72629	.95034	No
216.	243	2.65983	.95386	No
217.	1200	2.63558	.95511	No
218.	1816	2.57138	.95832	No
219.	1795	2.55110	.95931	No
220.	159	2.49874	.96179	No
221.	777	2.43879	.96452	No
222.	361	2.43125	.96486	No
223.	45	2.32675	.96932	No
224.	737	2.27760	.97130	No
225.	1548	2.25031	.97236	No
226.	221	2.18960	.97464	No

227.	975	2.18104	.97495	No
228.	550	2.01263	.98062	No
229.	1403	1.98124	.98158	No
230.	1906	1.96959	.98193	No
231.	1407	1.91224	.98359	No
232.	1010	1.86286	.98493	No
233.	1763	1.83625	.98563	No
234.	478	1.80112	.98651	No
235.	1248	1.73753	.98803	No
236.	593	1.70651	.98873	No
237.	418	1.68005	.98930	No
238.	1900	1.66402	.98964	No
239.	1920	1.65708	.98978	No
240.	1343	1.63393	.99025	No
241.	1384	1.63318	.99027	No
242.	717	1.54272	.99198	No
243.	1144	1.52562	.99227	No
244.	1615	1.51062	.99253	No
245.	1081	1.45507	.99343	No
246.	730	1.44582	.99357	No
247.	461	1.43681	.99371	No
248.	607	1.42623	.99387	No
249.	328	1.39269	.99435	No
250.	913	1.34058	.99505	No
251.	927	1.29106	.99566	No
252.	216	1.14305	.99717	No
253.	198	.98784	.99832	No
254.	1597	.95427	.99852	No
255.	1196	.90020	.99880	No
256.	1249	.58804	.99975	No
257.	44	.54880	.99981	No
258.	1813	.54493	.99982	No

Residuals Statistics

	Minimum	Maximum	Mean	Std. Deviation	N
Mahal. Distance	.596	55.093	7.969	6.558	258
Cook's Distance	.000	.065	.004	.007	258
Centered Leverage Value	.002	.214	.031	.026	258

Univariate outlier detection using z-score

Serial	Study member ID	Z-score of Total MET min/week	Outlier
259.	194	4.77007	Yes
260.	671	4.77007	Yes
261.	1192	4.16500	Yes
262.	832	3.25740	Yes
263.	1586	3.03050	Yes
264.	600	2.93325	No
265.	1621	2.89003	No
266.	1386	2.80359	No
267.	1875	2.80359	No
268.	1167	2.73877	No
269.	954	2.47945	No
270.	881	2.09048	No
271.	946	1.98675	No
272.	896	1.89599	No
273.	1269	1.89599	No
274.	205	1.83116	No
275.	1382	1.83116	No
276.	1774	1.83116	No
277.	1782	1.83116	No
278.	160	1.79875	No
279.	976	1.74472	No
280.	244	1.63668	No
281.	1876	1.63668	No
282.	675	1.59346	No
283.	1772	1.57185	No
284.	790	1.50702	No
285.	229	1.47460	No
286.	280	1.44219	No
287.	161	1.28012	No
288.	243	1.06402	No
289.	1149	1.05322	No
290.	1016	.98839	No
291.	1388	.98839	No
292.	1823	.98839	No
293.	720	.92356	No
294.	1679	.92356	No
295.	784	.90195	No
296.	221	.83712	No
297.	286	.83712	No
298.	1833	.79390	No
299.	1887	.68585	No
300.	660	.53459	No
301.	1526	.53459	No

302.	550	.50217	No
303.	1152	.46976	No
304.	1527	.46976	No
305.	1511	.44815	No
306.	1201	.40493	No
307.	1246	.38332	No
308.	459	.36171	No
309.	1374	.34010	No
310.	1144	.31849	No
311.	231	.30769	No
312.	825	.30769	No
313.	1560	.28608	No
314.	1585	.28608	No
315.	1200	.27527	No
316.	1343	.27527	No
317.	1814	.27527	No
318.	245	.25366	No
319.	1161	.23205	No
320.	1691	.23205	No
321.	1916	.21044	No
322.	1924	.21044	No
323.	232	.18883	No
324.	1403	.18883	No
325.	228	.16722	No
326.	1346	.16722	No
327.	202	.15642	No
328.	285	.14561	No
329.	593	.14561	No
330.	1834	.14561	No
331.	1902	.14561	No
332.	1920	.14561	No
333.	393	.11320	No
334.	1347	.10239	No
335.	147	.09159	No
336.	200	.08078	No
337.	289	.08078	No
338.	865	.08078	No
339.	1151	.08078	No
340.	1384	.08078	No
341.	1780	.08078	No
342.	1248	.05918	No
343.	639	.03757	No
344.	159	.01596	No
345.	693	.01596	No
346.	1258	.01596	No
347.	1795	.01596	No

348.	1360	-.02294	No
349.	1345	-.02726	No
350.	37	-.04887	No
351.	418	-.04887	No
352.	1006	-.09209	No
353.	404	-.10290	No
354.	610	-.11370	No
355.	676	-.11370	No
356.	903	-.11370	No
357.	980	-.11370	No
358.	1142	-.11370	No
359.	1143	-.11370	No
360.	1174	-.11370	No
361.	1196	-.11370	No
362.	1249	-.11370	No
363.	1742	-.11370	No
364.	1906	-.11370	No
365.	214	-.12451	No
366.	777	-.14612	No
367.	216	-.15692	No
368.	596	-.15692	No
369.	603	-.15692	No
370.	1518	-.15692	No
371.	795	-.17853	No
372.	948	-.17853	No
373.	1548	-.17853	No
374.	1569	-.17853	No
375.	1614	-.17853	No
376.	700	-.20014	No
377.	975	-.20014	No
378.	1357	-.20014	No
379.	1587	-.22175	No
380.	1881	-.22175	No
381.	198	-.24336	No
382.	212	-.24336	No
383.	328	-.24336	No
384.	692	-.24336	No
385.	1274	-.24336	No
386.	1895	-.24336	No
387.	1903	-.24336	No
388.	1827	-.27577	No
389.	461	-.28658	No
390.	299	-.28874	No
391.	889	-.30819	No
392.	913	-.30819	No
393.	1545	-.30819	No

394.	1615	-.30819	No
395.	1622	-.30819	No
396.	1669	-.30819	No
397.	193	-.32115	No
398.	259	-.32980	No
399.	352	-.32980	No
400.	652	-.32980	No
401.	1692	-.32980	No
402.	1767	-.32980	No
403.	659	-.34060	No
404.	1813	-.34060	No
405.	1935	-.34060	No
406.	44	-.37302	No
407.	282	-.37302	No
408.	412	-.37302	No
409.	607	-.37302	No
410.	1395	-.37302	No
411.	1563	-.38382	No
412.	166	-.39463	No
413.	1804	-.39463	No
414.	1831	-.40543	No
415.	310	-.41624	No
416.	767	-.41624	No
417.	1536	-.41624	No
418.	473	-.43785	No
419.	963	-.43785	No
420.	1597	-.43785	No
421.	1826	-.43785	No
422.	1904	-.43785	No
423.	717	-.45946	No
424.	730	-.45946	No
425.	1513	-.48106	No
426.	571	-.49187	No
427.	669	-.49187	No
428.	981	-.49187	No
429.	681	-.50267	No
430.	1538	-.50267	No
431.	1541	-.50267	No
432.	1796	-.50267	No
433.	705	-.52428	No
434.	882	-.52428	No
435.	1251	-.53509	No
436.	760	-.54589	No
437.	713	-.56750	No
438.	960	-.56750	No
439.	1081	-.56750	No

440.	1098	-.56750	No
441.	1423	-.56750	No
442.	1922	-.56750	No
443.	235	-.61072	No
444.	1188	-.61072	No
445.	1407	-.62153	No
446.	47	-.63233	No
447.	206	-.63233	No
448.	388	-.63233	No
449.	927	-.63233	No
450.	1342	-.63233	No
451.	48	-.66475	No
452.	746	-.66475	No
453.	197	-.67555	No
454.	219	-.67555	No
455.	376	-.67555	No
456.	1900	-.67555	No
457.	1901	-.67555	No
458.	233	-.69716	No
459.	361	-.69716	No
460.	738	-.69716	No
461.	1359	-.69716	No
462.	1537	-.69716	No
463.	32	-.71877	No
464.	737	-.71877	No
465.	747	-.71877	No
466.	1544	-.71877	No
467.	1707	-.74038	No
468.	1816	-.74038	No
469.	38	-.75118	No
470.	478	-.76199	No
471.	611	-.76199	No
472.	1435	-.76199	No
473.	1543	-.76199	No
474.	1914	-.76199	No
475.	31	-.78360	No
476.	657	-.78360	No
477.	677	-.78360	No
478.	1189	-.78360	No
479.	1245	-.78360	No
480.	1558	-.79440	No
481.	664	-.80521	No
482.	1010	-.80521	No
483.	1011	-.80521	No
484.	1581	-.80521	No
485.	1589	-.80521	No

486.	79	-.82682	No
487.	735	-.82682	No
488.	1184	-.82682	No
489.	1590	-.82682	No
490.	34	-.87004	No
491.	46	-.87004	No
492.	49	-.87004	No
493.	574	-.87868	No
494.	50	-.88084	No
495.	709	-.88084	No
496.	45	-.89165	No
497.	347	-.89165	No
498.	793	-.89165	No
499.	1925	-.89165	No
500.	1936	-.89165	No
501.	1763	-.91326	No
502.	565	-.92406	No
503.	365	-.93487	No
504.	672	-.93487	No
505.	716	-.94351	No
506.	35	-.95648	No
507.	398	-.95648	No
508.	1198	-.95648	No
509.	486	-.99970	No
510.	1809	-.99970	No
511.	869	-1.00834	No
512.	1905	-1.02130	No
513.	43	-1.06452	No
514.	196	-1.10774	No
515.	680	-1.10774	No
516.	1736	-1.15096	No
