# Supplementary material for: Healthy behaviours, treatment, and control status of diagnosed hypertension and diabetes among the government nurses and para-health professionals of Bangladesh: A cross-sectional study
Source: PLOS Glob Public Health. 2023 Aug 1;3(8):e0002234. doi: 10.1371/journal.pgph.0002234 (PMC10393152; doi:10.1371/journal.pgph.0002234)
Supplement: Attachment — Submitted filename: Response to Reviewers.pdf [file pgph.0002234.s002.pdf]

Reply to editor & reviewers

Reply to editor

Dear academic editor (Sherly Parackal), many thanks for your valuable comment. Here we have explained regarding your concern.

Comment 1

The questions used to collect data on healthy behaviors (smoking etc.) to control hypertension and diabetes. Did the questions explicitly ask if these behaviors were undertaken to control hypertension and diabetes

Reply

No, we did not explicitly ask if they tried to modify the behaviors to control hypertension and diabetes. We thought if we asked explicitly, then the findings could be biased as they knew about the control measures as a health professional and, risk factors-specific questions leaded them to a common positive answer. We assumed as a health professional, their knowledge, attitude and practice about the risk factors and chronic diseases are different than the general population. We collected information about the risk factors and chronic diseases from all the health professionals. For current study, their awareness about hypertension & diabetes was evaluated in terms of their screening habit (first two variables of Table 1 & table 2). The distribution of risk factors was evaluated among those diagnosed as hypertensive and diabetic (third variable of Table 1 & 2) while they screened themselves at any point of their life (screened at least once in their lifetime) and found themselves with the disease. Then their healthy lifestyle practice was presented as 'Yes' meaning 'used to practice' or 'No' meaning 'not used to practice' according to global recommendations for each risk factors (foot notes of Table 1 & Table 2). Risk factors distribution beyond the recommendation indicated they were not adhering to healthy lifestyle practice being a diabetic or hypertensive.

Comment 2

The questions used to collect data on "days since the last measurement of HBP and BG". Were these open-ended questions? If categorical what where the categories used?

Reply

It was an open-ended question.

Reply to reviewer 1

Dear respected reviewer, thanks for your valuable comment. Here we have provided reply to your comments.

Comment 1

Introduction

Page 3, Line68: In this study, the authors assessed only hypertension and diabetes. Why did the author focus on these two diseases rather than other lifestyle-related diseases or mental health which has been often reported among health professionals? An explanation regarding this part of the study would support the significance of this study.

Reply

We agree with your comments that other than diabetes and hypertension there are many other NCDs. But Bangladesh perspective it is completely different. First, here hypertension and diabetes are mostly assessed NCD and evaluated in various settings including various population. However, these two midstream/intermediate risk factors have not yet been evaluated among health professionals. Hence, we targeted these two risk factors to assess the distribution among the health professionals. Second, these two risk factors are easy to address than other NCDs and routinely assess in all healthcare settings. Our participants were recruited by the higher authorities of Government to participate in a training session lead by the authors. They were not intended to participate in a study. With the help of Government officials, we invited them to participate as a study population and they agreed. As there was no fund for the study, it was not possible for us to assess other risk factors/NCDs which needed objective assessment like biochemical measurement to address dyslipidemia etc. Again, we needed to adopt an assessment that consumed relatively less time/duration for the participants. Assessment of various aspect of mental health need valid questionnaire which are mostly long form. We have added some new evidences based on our country and global literatures in response to the comment:

Page number 03, line number 67-72

"*In Bangladesh, cardiovascular disease (CVD) is the predominant and downstream cause of mortality from chronic diseases [7]. HTN and DM are the two main intermediate risk factors of CVD which have higher burden and relatively easy to screen in a low resource setting. Although easy to detect and prevent, previous research found a notable prevalence of these two risk factors among health professionals in Bangladesh [4,8]. Global research also reported higher prevalence of HTN and DM among the health professionals [9,10,11,12,13]*"

Comment 2

METHODS

Page 3, Line 75: Please explain SSNs. Are SSNs certified qualifications?

Reply

It is a government authorized position offered based on seniority, experience and also qualification (Diploma/BSc).

Comment 3

DISCUSSION

Page 8, Line 185: Author discussed that "It seems that HPs neglected these risk factors or hold idealistic view of their role in treating and fighting diseases." What does the meaning of hold idealistic view of their role in treating and fighting diseases? Please explain to make the readers understand easily.

Reply

The sentence was used to reflect that the health professionals try to maintain an image of invulnerability to the general population. This is because they treat and look after the well-being of their patients. As they are the 'care giver', they most often try to hide their health issues, consider self-prescription and thus neglect their own health. As chronic disease has long latent period to develop, such long-term self-negligence led to final expression of diseases. We have added information as per the suggestion of reviewer:

Page-09, line-195 to 196

"Their idealistic view is that they are a 'caregiver', not 'care taker'. Hence, they rarely seek treatment from other health professional [26]"

Comment 4

There were no differences between SSNs and PHPs, except for smoking. What do you consider these results? Were these results as you hypothesized? Please discuss about that.

Reply

Yes, it exactly what we assumed. This is because in Bangladesh SSN usually represent women gender than men. In our study we also found SSNs=87.8% women, 12.2% men; PHPs=85.3% men, 14.7% women. Again, women are not usually smoker in Bangladesh. Thus, the gender made a significant difference statistically in our paper. We have already discussed it in our 1st submission:

Page-09, line number-199 to 205

"We found that avoidance of smoking is the only risk factor significantly associated with health professional categories. However, possibly it elucidated due to the influence of gender (SSNs=87.8% women, 12.2% men; PHPs=85.3% men, 14.7% women). This gender-based association with smoking has already been reported by a study among HPs in Bangladesh [22]. In Bangladesh, culturally and religiously, women are not generally smokers and working in the health sector also reduces the possibility of being a smoker."

Comment 5

Based on the results, how should health professionals be educated to maintain their health?

Reply

We believe health professionals had knowledge about healthy lifestyle. But the problem is in their attitude towards chronic diseases and their own health. We described it as an 'idealistic view'. So, we need to motivate them to maintain healthy recommended lifestyle. We suggested to focus mental health as part of health education intervention and this issue has already mentioned in the conclusion part:

Page-09 to 10, line-223 to 227

"For this, attitude towards chronic diseases and their own health should be changed as they are expected to be caregivers and not "caretakers". Hence, policy makers should develop separate screening and health education intervention packages including mental health for HPs to motivate them and allow to lead an active and a productive life"

Reply to reviewer 2

Dear respected reviewer, thanks for your valuable intellectual comment. Here we have provided reply to your comments.

Comment 1

Abstract: The abstract of the paper is too long. At the moment, it seems to be a part of introducing. The abstract should be concise and summarize the entire paper.

Reply

The journal allowed 300 words in the abstract section. However, our abstract was 281 words. We believe our presentation was a summarized form of full manuscript that followed journal allocated word count. Hence, we have not brought any change.

Comment 2

Introduction: The author(s) need to better motivate the study by adding what is already known about this topic in Bangladesh. Further, it would be appropriate to mention the key contributions of this study and how they differ from existing literature in Bangladesh.

Reply

These issues have already mentioned in our first submission. Here we explained:

Already known about this topic in Bangladesh: In our first submission we stated what has already known about this topic (page 3, line 54 to 57)-

"In case of health professionals, the scenario may be different as previous study found a high prevalence of hypertension (HTN), diabetes mellitus (DM) and other NCD risk factors among the health professionals of Bangladesh [4]. However, their healthy behavioral practice has not yet been evaluated in any context that needs to be explored."

In our revised submission, we also added few information (page 3, line 66 to 72)-

"In Bangladesh, cardiovascular disease (CVD) is the predominant and downstream cause of mortality from chronic diseases [7]. HTN and DM are the two main intermediate risk factors of CVD which have higher burden and relatively easy to screen in a low resource setting. Although easy to detect and prevent, previous research found a notable prevalence of these two risk factors among health professionals in Bangladesh [4,8]. Global research also reported higher prevalence of HTN and DM among the health professionals [9,10,11,12,13]."

Key contributions of this study and how they differ from existing literature in Bangladesh:

First time, we reported the pattern of healthy behavioral practice to control hypertension and diabetes among government health professionals of Bangladesh. So, there is no such evidence in existing literatures for health professionals in Bangladesh to compare and also to cite. However, existing literature reported their working environment that prone them to develop chronic condition which we have already mentioned in our first submission: page 3, line 61 to 64

"Countries like Bangladesh, most of them deliver health services in a low-resource settings with disproportionate distribution of skilled workforce. So, they adapt themselves physically and mentally in an overburdened, understaffed, and insufficiently equipped health care setting that puts unusual stress [6]"

Comment 3

3. Material and Methods:

a. The author(s) should use either "a survey or questioner" in the data collection part.

Reply

We used questionnaire.

b. What is the motivation for using the sample only from the capital city, Dhaka?

Reply

We did not use sample from Dhaka. Our study site was at Dhaka. But sample from all Upazila Health Complexes in the country covering 8 divisions and 64 districts. The sample of health professionals were sent by the Non-communicable Disease Control cell of the Directorate General of Health Services to participate in a 3-day training on NCD management. The training was conducted at a Medical University situated in Dhaka city.

c. The author(s) discussed in the abstract that the sample is from the capital city, Dhaka. However, the author(s) did not discuss it in the paper. Why is it so?

Reply

It seems the comment 'b' and 'c' are similar. Hence, the aforementioned explanation is also applicable for this comment.

d. There is no information about the information sheet. Is it provided before the survey?

Reply

We have added the information in page 4, line 98- *"The questionnaire was distributed on the first day of the training"*

e. Please provide details on the "Declaration of Helsinki (October 2013)."

Reply

Here, the "Declaration of Helsinki (October 2013)" is not part of the manuscript or reflected any objective that measured by the investigators. We mentioned it to reflect higher ethical standard followed by this research for human subjects. It is a statement of ethical principles for medical research involving human subjects. This is the journal requirement to provide a statement that we followed "Declaration of Helsinki (October 2013)." We are suggesting the respected reviewer to follow the journal link to know more about it: https://journals.plos.org/globalpublichealth/s/submission-guidelines

The authors don't want to unusually increase the volume of the paper to write about "Declaration of Helsinki (October 2013)."

f. Can you please explain "residual confounders"? Unfortunately, it is not a common term for healthcare research.

Reply

Here 'residual confounder' means uncontrolled/unadjusted confounder. It has two components: (i) unmeasured confounder (ii) incorrect analysis. In out tables, in the final model of logistic

regression, we adjusted all the variables that were screened using univariate analysis. As we adjusted all the variables in the final model, so there was no unmeasured confounder exist. Thus, the possibility of an effect of residual confounder was eliminated.

Comment 4

Results:

a) The result section is more of a statistical exercise, where the author(s) differentiate based on different statistics. Therefore, the author(s) should explain it more simply so the reader can easily understand it.

Reply

Apologize if you misunderstand. This is because we did not find anything in our result section that was difficult to read or interpret. Our presentation used a format that commonly appears in scientific journals. We mentioned only four statistical term which are very common in the scientific journals: mean & standard deviation, percentages/proportion, adjusted odds-ratio (AOR) & p-value.

We will be glad if the respected reviewer explicitly mention which part is difficult to interpret or the way how the readers will be benefitted more than the current format which is usual in scientific journal.

b) The paper has two tables (Table 1 and Table 2). However, none of these is referred to anywhere in the paper.

Reply

Obviously, this was a great mistake. Now we have added. Thank you again.

c) Results need to be discussed in more detail and explained in easy words so the audience can understand them better.

Reply

We believe results should be discussed in more details in ‘Discussion’ part of manuscript. Moreover, duplication of results in the text (same to same) from the Table is not permitted in scientific journal as per our knowledge. We are requesting to follow the aforementioned reply against the “*Comment 4 (a)*”.

Comment 5

Discussion: Most references are just cited in the discussion section. The author(s) should adequately link the cited papers in the discussion. The author(s) should state whether their results align with the prior literature or differ from theirs. It is good to write the discussion by looking at the similarities and differences between the literature and the strength and limitations.

Reply

This form of generalized comment difficult to address while we have already presented the discussion (in 1st submission) as the reviewer suggested. However, we tried to add some similarities and differences in our revised version where appropriate:

In revised version, more literatures have cited in the “*Introduction*” part of manuscript.

1st evidence (HPs self-screening practice)-we discussed the similarities in our first submission. In revised version, we also added the difference in terms of 'health care facilities-primary or tertiary'.

Page 06, line 154-155

"Although both evidences [18,19] aligned with our findings, they studied among the health professionals rather than the primary care"

In our first submission, we also explained why we found such evidence (Page 06, line 156 to 161)

2nd evidence (prevalence of HTN was higher than the DM among those screened)-in our first submission we compared with a nationally representative study that followed same trend. In revised version, we also added that the nationally representative study did not include HPs as a study population and this is the difference from current study. We also added another study to support the findings (Page 06, line 162-164)

3rd evidence (treated for HTN and DM; and control rate)- in our first submission we compared with another study that found same findings. The control rate was also compared with international findings. We believe it was sufficient (page 06, line 166-170)

4th evidence (uncontrolled risk factors)-we explained why HPs did not practice healthy behaviors up to recommended level with supporting reference (page 9, line 193-199)

5th evidence (smoking showed significant difference)- we explained why such difference found for HPs with supported evidence from Bangladesh (page 9, line 199-205)

Comment 6

Author should also add the limitations of the study.

Reply

In our first submission, we discussed about our limitation in the page number 09, line number 214-216.
