# Supplementary material for: Healthy behaviours, treatment, and control status of diagnosed hypertension and diabetes among the government nurses and para-health professionals of Bangladesh: A cross-sectional study
Source: PLOS Glob Public Health. 2023 Aug 1;3(8):e0002234. doi: 10.1371/journal.pgph.0002234 (PMC10393152; doi:10.1371/journal.pgph.0002234)
Supplement: Attachment — Submitted filename: Response to Reviewers.pdf [file pgph.0002234.s003.pdf]

Reply to editor & reviewers

Reply to editor

Dear academic editor (Sherly Parackal), many thanks for your valuable comment. Here we have explained regarding your concern.

Comment 1

Overall: Please improve the written language of your manuscript and check for grammatical errors and sentence structures. For e.g. Chronic illness among health professionals is merely reported. Using the word rarely would better convey what you are trying to communicate. There are several other sentences that require revising for achieving good written standard.

Reply

We have changed accordingly. We brought change in the title also. We tried our best to address grammatical issues.

Comment 2

Abstract: Please avoid repetitions for e.g. 100% sample. This should be referred to as a census rather than a 100% sample.

Reply

We have changed as suggested.

Line number: 29-30

Comment 3

Methods: Please include clearly the methods adopted for measuring HTN and diabetes and anthropometry. The references and standards are good but readers need to know how this was done. How many measurements/readings were taken. Were any bias in measurements introduced by different people taking measurements. Who took the measurements, where they trained?

Reply

- Physical measurement has added in details (line 93-108).
- Three investigators who were the chief trainer and authors of the manuscript measured the anthropometry, blood pressure and blood glucose separately. One investigator measured height and weight, others measured blood pressure and blood glucose respectively. As the investigators were experienced and individually measured only one parameter, the chance of measurement error and bias was less.

Comment 4

Objectives and results: your objective-risk factors modification for diagnosed cases is incorrect as you did not ask this question. Your explanation ...No, we did not explicitly ask if they tried to modify the behaviors to control hypertension and diabetes. We thought if we asked explicitly, then the findings could be biased as they knew about the control measures as a health professional and, risk factors-specific questions leaded them to a common positive answer. We assumed as a health professional, their knowledge, attitude and practice about the risk factors and chronic diseases are different than the general population.... is not acceptable. Please change the wording of this aim, in the tables, results and discussion. What would be more appropriate is "assessing healthy behaviours among those diagnosed with diabetes and HTN". Could the lack of key healthy behaviours among those diagnosed be a reason for poor control of HTN and diabetes? Since you did not ask the question whether they practiced these healthy behaviours for controlling diabetes or HTN, you need to rephrase your sentences accordingly. For e.g. Results: Line 141: Regarding healthy lifestyle practice to control HTN and DM... should be rephrased. As per Table 1: only 63.4% had a controlled HTN and Table 2: 31% had controlled diabetes. Your analysis includes all those who were diagnosed with HTN and diabetes, hence you cannot say that these healthy behaviors were practiced for controlling HTN or diabetes.

Reply

Thanks for your intellectual input. We considered all the suggestions and brought changes in the following sections:

- Title: Healthy behaviours, treatment, and control status of diagnosed hypertension and diabetes among the government nurses and para-health professionals of Bangladesh: A cross-sectional study
- Objective: "*Therefore, this study aimed to assess health behaviours, treatment, and control status among government-employed health professionals who were diagnosed with DM and HTN.*"
- Tables: Title and subheading

Table 1: Healthy behaviours, treatment, and controlled status of diagnosed HTN among the SSNs and PHPs of Bangladesh (N=1912)

Table 2: Healthy behaviours, treatment and controlled status of diagnosed DM among the SSNs and PHPs of Bangladesh (N=1912)

Changed sub-headings for both tables: ‘Practice healthy behaviours to control HTN and DM’ changed to ‘Healthy behaviours’

- Results (line 154): “Regarding healthy behaviours among those diagnosed with HTN and DM”
- Discussion (line 194-199): “*We found a high prevalence of key risk factors among those diagnosed with HTN and DM namely physical inactivity, unhealthy diets (low fruit and/or vegetable intake and high salt intake), and overweight/obesity. The possibility of such high prevalence includes a lack of self-motivation and negligence. Regarding self-motivation, around 26% of doctors with medical problems reported feeling inhibited to seek treatment [25].*”

Comment 5

You need to expand on the limitations of the study. Apart from recall bias due to the self reported nature of the questionnaire data are there any other limitations? for e.g. in measuring HTN and diabetes or anthropometry? What about missing data and outliers? For e.g. your METS per week data has substantial outliers. How did you deal with this? You have reported this data as median, why?

Reply

- We have included possible limitations (line 240-246)
- We checked outliers using multivariate outlier detection method namely ‘The Mahalanobis Distance (MD) Method’ & interpret it using corresponding *p*-value (<0.001 indicated the outlier). In this regard a supplementary file has added for evaluation. Again, the influence of the identified outliers was evaluated using the ‘Cook's distance’. The Cook’s distance is considered extreme if it is greater than 1. If a point has been flagged by the Cook’s distance, the point is considered highly influential and has a combination of unusual explanatory variables and response value. We found 03 outliers using multivariate outlier detection method (Mahalanobis Distance Method). However, those outliers had no impact on our analysis based on ‘Cook’s distance’.
- For evaluation of MET value, we used univariate outlier detection method namely ‘Z-score’. In this case, z-score greater than +3/-3 was considered as an outlier. We found 05 outliers of MET value. However, aforementioned multivariate outlier evaluation using regression did not find any influential data points using the Cook’s distance. Hence, we did not remove them.
- We did not report our MET value using median in any part of our manuscript. If we are wrong, kindly specify using line number.

- A total of 30 responses out of 1942 were found incomplete (missing) or inconsistent, and hence we removed them (line number 141-142).

Reply to reviewer 1

Dear respected reviewer, thanks for your valuable comment. Here we have provided reply to your comments.

Comment 1

The reviewer asks the authors to discuss how they believe that the lack of differences between SSNs and PHPs was not due to differences in gender, but rather the lack of differences in job type.

The authors answered that SSNs is a government authorized position offered based on seniority, experience and also qualification (Diploma/BSc).

Do SSNs and PHPs have the same level of health care knowledge and skills?

Readers unfamiliar with the health care system and the education system in Bangladesh may be confused by this discussion.

For this reason, I pointed out that the authors should explain what SSNs is and how they differ from PHPs in the first peer review.

I am sorry, but since there is no discussion on this, I cannot support the education for the health care profession that the author states in his current conclusion.

We believe it is important in your paper to explain and discuss the situations in Bangladesh for the benefit of readers who are unfamiliar with it.

Reply

We have provided our explanation in the discussion part (line number 174-184) as follows:

"We found job-specific differences in the domain of treatment and control which was statistically non-significant. Here, the rate for treatment and control of HTN was higher in SSN whereas DM treatment and control were higher among PHPs. The exact cause of such differences among HPs based on their profession category is not known. Although they are working in the same healthcare setting, possibly their knowledge, attitude and practice do not converge [4]. This is because the educational qualification and clinical training of SSN (Diploma/BSc/MSc) is completely different from the PHPs (Diploma) and their clinical role is also varied in Bangladesh. Here, SSNs were mostly involved in indoor patient care, and Sub-assistant community medical officers (SACMO) were provided treatment in primary care settings as an assistant to qualified doctors. On the other hand, medical technicians (MTs) and sanitary inspector had a very limited role in direct patient management [4]."
